# Benzoyl Peroxide Oxidation Route to the Synthesis of Solvent Soluble Polycarbazole

**DOI:** 10.1155/2014/987236

**Published:** 2014-10-29

**Authors:** Rajender Boddula, Palaniappan Srinivasan

**Affiliations:** ^1^Polymers & Functional Materials Division, Indian Institute of Chemical Technology, Hyderabad, Telangana 500007, India; ^2^CSIR, Network Institutes for Solar Energy (NISE), India

## Abstract

Carbazole was oxidized by benzoyl peroxide in presence of *p*-toluenesulfonic acid to polycarbazole salt at room temperature for the first time. Polycarbazole salts were synthesized via solution and emulsion polymerization pathways. Polycarbazole bases were prepared by dedoping from polycarbazole salts. Formation of polycarbazoles was confirmed from infrared, electronic absorption and EDAX spectra. Polycarbazole salt was obtained in amorphous nature in semiconductor range (10^−5^ S/cm), which was found to be soluble in less and high polar solvents. Polycarbazole salt prepared by emulsion polymerization pathway showed mixture of shapes with microrod, sphere, and pores, whereas its corresponding base showed only micropores structure. On the other hand, polycarbazole salt and its corresponding base prepared by solution polymerization pathway showed flake-like morphology. Higher thermal stability was obtained for polycarbazole salt prepared by emulsion polymerization pathway than that of the salt prepared by solution polymerization pathway.

## 1. Introduction

Conductive polymers or, more precisely, intrinsically conducting polymers are organic polymers that conduct electricity [1], and such compounds may have metallic conductivity or can be semiconductors. Some of the well-studied organic conductive polymers, according to their composition, are polyaniline, polypyrrole, polythiophene, polyacetylene, poly(p-phenylene vinylene), poly(p-phenylene sulfide), and polycarbazole.

Polycarbazole (PCz) and its derivatives, which have a structure of pyrrole ring with two fused benzene rings, are well known to exhibit good electroactive and photoactive properties [2]. PCz is being explored for various applications especially in hole transporting and photoluminescence efficiency, electroluminescence [3–5], light-emitting diodes [6], electrochromic displays [7], supercapacitor [8], chemical batteries [9, 10], sensors [11, 12], and laser dyes and organic transistors [13].

Studies on polycarbazole and its derivatives synthesized by electrochemical method have been reported [14–17]. However, work on chemical synthesis of unsubstituted carbazole is very few, even though chemical synthesis for substituted polycarbazole had already been reported [18–20]. Chemical synthesis of PCz is drawing keen attention due to its obvious advantage in morphology control and bulk synthesis. In 2010, first report of chemical synthesis of unsubstituted PCz in hollow microspheres morphology was reported by Gupta and Prakash [21] and Raj et al., that is, the synthesis of soluble PCz by the chemical oxidative polymerization of carbazole using ammonium persulfate in acetonitrile medium [5]. Recently, PCz-Au composite was synthesized by the oxidation of carbazole using HAuCl_4_ oxidant [22].

There is a restriction on the selection of the oxidizing agents due to nonsolubility of oxidizing agents and monomers in the same solvent. This problem aggravates in case of carbazole as it is insoluble in aqueous media. Since carbazole is not soluble in water and soluble in chloroform, carbazole was oxidized using chloroform soluble benzoyl peroxide oxidant by solution polymerization pathway. Also, carbazole was polymerized to polycarbazole, that is, emulsion polymerization pathway using sodium lauryl sulfate as emulsifier. Polycarbazole was characterized by physical, electrical, spectral, and thermal methods.

## 2. Experimental

### 2.1. Instruments and Characterization

Powder of polycarbazole was pressed into a disk of 13 mm diameter and about 1.5 mm thickness under a pressure of 120 Kg/cm^2^. Resistance of the pellet was measured by two-probe method using 2010 digital multimeter (Keithley, Cleveland, Ohio, USA). The conductivity of the pellet was calculated by the formula *σ* = *l*/(*A*∗*R*), where, *l* = thickness of the pellet in cm, *A* = area of the pellet in cm^2^, and *R* = resistance of the pellet in Ω. Pellet density was measured from mass per unit volume of the pressed pellet using the formula *d* = *m*/(*πr* ^2^ *l*), where *l* = thickness of the pellet in cm and *m* = mass of the pellet in g. Molecular weight of polycarbazole was carried out using MALDI-TOF-MS instrument (Shimadzu Biotech Axima Performance 2.9.3.20110624: Model Linear, Power: 80, Blanked, P. Ext@4300 (bin 97) UK) using *α*-cyano 4-hydroxy cinnamic acid matrix. FT-IR spectra of polymer samples were registered on a FT-IR spectrometer (Thermo Nicolet Nexus 670, USA) using the KBr pressed pellet technique. X-ray diffraction profiles for polymer powders were obtained on a Siemens/D-500 X-ray diffractometer, USA, using Cu K*α* radiation and scan speed of 0.045°/min. Morphology studies (microstructural and elemental analyses) of the polymer samples were carried out with a Hitachi S-4300 FE-SEM (Tokyo, Japan). The sample was mounted on a carbon disc with the help of double-sided adhesive tape and sputter-coated with a thin layer of gold to prevent sample charging problems. Thermogravimetric analyses of polymer samples were carried out using TA Instruments (TGA Q500 V20.8, USA) from ambient to 700°C under nitrogen atmosphere at a heating rate of 10°C per minute. Cyclic voltammetry experiment was carried with a WonATech multichannel potentiostat/galvanostat (WMPG 1000, Gyeonggi-do, Korea).

### 2.2. Preparation of Polycarbazole Salt (S-PCz-*p*-TSA) by Solution Polymerization Pathway

In a typical polymerization system, carbazole (1.67 g, 0.1 M), and* p*-toluene sulfonic acid (3.8 g, 0.2 M ) were dissolved in 50 mL of chloroform and magnetically stirred for 30 min. To this solution, 50 mL of chloroform solution containing benzoyl peroxide (3.6 g, 0.24 M) was added as a whole. The mixture was stirred constantly for 4 h at an ambient temperature. The mixture was stirred constantly for 4 h at an ambient temperature and then precipitated with methanol. The green precipitate was filtered and washed several times with distilled water followed by methanol. The powder sample was dried at 50°C in an oven until a constant weight.

### 2.3. Preparation of Polycarbazole Salt (E-PCz-*p*-TSA) by Emulsion Polymerization Pathway

In a typical polymerization system, carbazole (1.67 g, 0.1 M) was dissolved in 50 mL of chloroform. 25 mL aqueous solution containing 3.8 g* p*-toluene sulfonic acid (0.2 M) was added while stirring the solution. Then 25 mL aqueous solution containing 1 g of SLS surfactant (0.035 M) was added slowly while stirring which resulted in an emulsion form. To this emulsion, 25 mL chloroform solution containing 3 g of benzoyl peroxide (0.1 M) was introduced. The mixture was stirred constantly for 4 h at an ambient temperature and then precipitated with methanol. The green precipitate was filtered and washed several times with distilled water followed by methanol. The powder sample was dried at 50°C in an oven until a constant weight.

### 2.4. Preparation of Polycarbazole in Base Form by Dedoping of Polycarbazole Salt

Polycarbazole (S-PCz or E-PCz) was prepared from polycarbazole salt prepared by solution or emulsion polymerization pathway by dedoping process. 0.5 g of polycarbazole salt was stirred in 50 mL of 1 M aq. NaOH solution for 4 h and then filtered and washed several times with distilled water followed by methanol. The powder sample was dried at 50°C in an oven until a constant weight.

### 2.5. Solubility of Polycarbazole

0.5 g of polycarbazole salt was stirred in 50 mL CHCl_3_ solvent for 4 h at an ambient temperature and then filtered and dried. Weight of the dried polycarbazole powder was taken. Amount of soluble portion was calculated from the weight difference.

E-PCz-*p*-TSA and S-PCz-*p*-TSA salts are almost completely soluble in THF, DMSO, and NMP solvents, whereas the solubility of E-PCz-*p*-TSA and S-PCz-*p*-TSA in chloroform is found to be 64 and 9 wt%, respectively. E-PCz and S-PCz bases are almost soluble in DMSO and NMP.

## 3. Results and Discussion

### 3.1. Preparation and Characterization of Polycarbazole

**Scheme 1:**
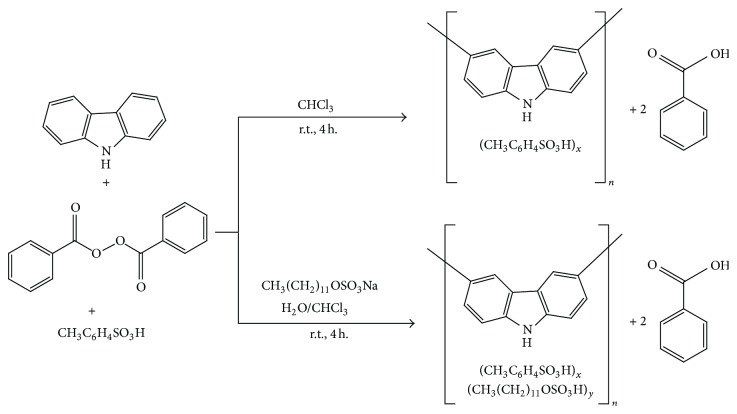
Synthesis of polycarbazole salts via solution (top) and emulsion (bottom) polymerization pathways.

For the first time, benzoyl peroxide is used as an oxidizing agent for the oxidation of carbazole to polycarbazole. Polycarbazole was prepared both by solution and emulsion polymerization pathways by oxidizing carbazole using benzoyl peroxide oxidant in the presence of acid and with or without sodium lauryl sulfate emulsifier (Scheme 1). Polycarbazole salts are dedoped to its corresponding polycarbazole.

Polycarbazole salt was prepared by solution polymerization by changing the concentration of acid, oxidant, and reaction time. The values of yield, conductivity, and densities of polycarbazole salts are reported in Table 1. The observations are as follows.

- The values of yield, conductivity, and densities of the polycarbazole salts prepared using 0.1 and 0.2 M of* p*-TSA are found to be nearly the same.
- Similarly, the values of yield, conductivity, and densities of the polycarbazole salts prepared in 4 h are found to be nearly the same with the sample prepared in 8 h. However, the value of yield increases, that of conductivity decreases, and that of density is found to be the same in 24 h in comparison with 8 h.
- The values of yield and conductivities of the polycarbazole salts increase with increase in the concentration of the oxidizing agent and the values decrease on further increase in concentration of oxidizing agent.
- Pellet density of polycarbazole salts is found to be the same (1.11 to 1.18 g/cm^3^)
- Maximum yield (69%) and conductivity (1.5 × 10^−5^ S/cm) of the polycarbazole salt were obtained with the reaction condition such as carbazole (0.1 M),* p*-TSA (0.2 M), BPO (0.12 M), and reaction time: 4 h.

For comparison, polycarbazole salt was also prepared by emulsion polymerization pathway using aqueous/nonaqueous media in presence of sodium lauryl sulfate surfactant. Yield of polycarbazole salt prepared by emulsion polymerization pathway using 0.2 M* p*-TSA protonic acid (60%) is found to be slightly higher than that of 1 M HCl (55%) used. Densities and conductivities of the polycarbazole salts could not be measured because of the course nature of the samples, which in turn did not give pellet.

### 3.2. Molecular Weight of Polymer

**Figure 1:**
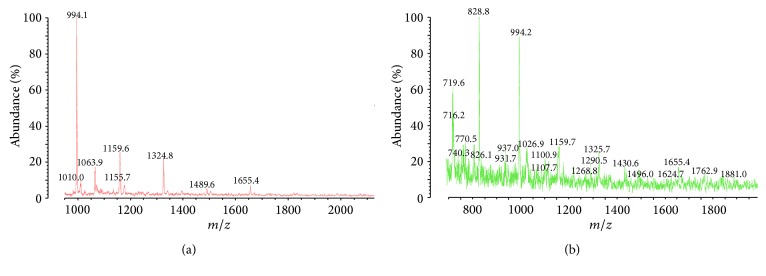
(a) MALDI-TOF mass spectrum of S-PCz-*p*-TSA and (b) MALDI-TOF mass spectrum of E-PCz-*p*-TSA.

Molecular weight of the polycarbazoles S-PCz-*p*-TSA and E-PCz-*p*-TSA was measured by MALDI-TOF-MS using *α*-cyano 4-hydroxy cinnamic acid matrix and is shown in Figures 1(a) and 1(b), respectively. Molecular weight of S-PCz-*p*-TSA and E-PCz-*p*-TSA was found to be 1655 and 2005, which corresponds to approximately 10 and 12 monomer units, respectively. However, this molecular weight corresponds only to the soluble part of polymer in THF isolated portion. Gupta and Prakash reported the molecular weight of polycarbazole prepared by interfacial polymerization method as 1971 (~12 monomer units) from GPC measurement [21].

### 3.3. Infrared Spectra of Polycarbazole

**Figure 2:**
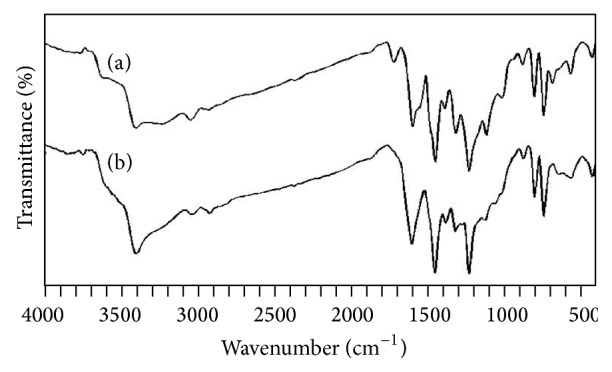
Infrared spectra of (a) S-PCz-*p*-TSA and (b) S-PCz.

**Figure 3:**
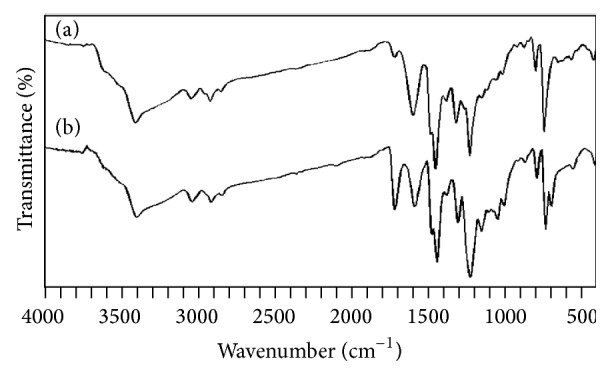
Infrared spectra of (a) E-PCz-*p*-TSA and (b) E-PCz.

The infrared spectra of polycarbazole salt and its corresponding base prepared by solution and emulsion polymerization pathways are shown in Figures 2 and 3, respectively. Assignments for the four polycarbazole samples are reported in Table 2.

Significant observations are as follows.

- A peak at 2925 cm^−1^ is observed in all the cases (both salt and bases) and this peak may be due to impurities or supertone band or shifted aromatic C–H str. frequency due to the presence of radical on nitrogen atom.
- A peak at 1730 cm^−1^ is observed for polycarbazole salts (E-PCz-*p*-TSA and S-PCz-*p*-TSA), whereas a small peak is observed forE-PCzand no peak for S-PCz. This peak may be often due to *δ*-OH from water adduct [5] or C=O str. due to overoxidation.
- Peaks at 1165 and 1060 cm^−1^ observed for polycarbazole salts (E-PCz-*p*-TSA and S-PCz-*p*-TSA) are due to sulfonic group, whereas the peaks almost vanished for polycarbazole bases (E-PCzand S-PCz). This result confirms the presence of* p*-TSA in polycarbazole salts as dopant.

### 3.4. Electronic Absorption Spectra of Polycarbazole

**Figure 4:**
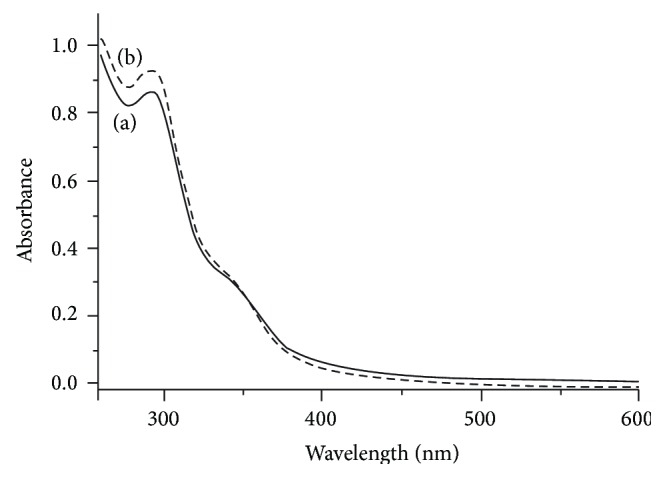
Electronic absorption spectra of (a) S-PCz-*p*-TSA and (b) S-PCz.

Polycarbazole salt and its corresponding base samples prepared by emulsion polymerization pathway were dissolved in dimethyl sulfoxide and then filtered to get a clear solution. DMSO solutions were subjected for UV-Vis spectral measurement at ambient temperature. Both the salt (E-PCz-*p*-TSA) as well as its base (E-PCz) showed one peak at 298 and another shoulder around 350 nm (Figure 4). An absorption band of the polymer at 298 nm corresponds to valance band to conduction band and a shoulder around 350 nm is due to polaron level to *π* ^*^ conduction band. These two peaks of the polymer (298 & 350 nm) are different from the monomer peaks of carbazole observed at 289 and 325 nm, respectively [21]. A similar UV-Vis spectrum was observed for polycarbazole salt and its corresponding base samples were prepared by solution polymerization pathway.

### 3.5. Energy Dispersive X-Ray Spectroscopy of Polycarbazole

Element of sulphur present in PCz systems was qualitatively found out from energy dispersive X-ray spectroscopy. EDAX results showed that polycarbazole salt contains sulphur elements S-PCz-*p*-TSA (1.85%) and E-PCz-*p*-TSA (1.73%), whereas polycarbazole base does not contain sulfur and this result supports the presence of* p*-TSA as dopant in polycarbazole salt.

### 3.6. FE-SEM of Polycarbazole

**Figure 5:**
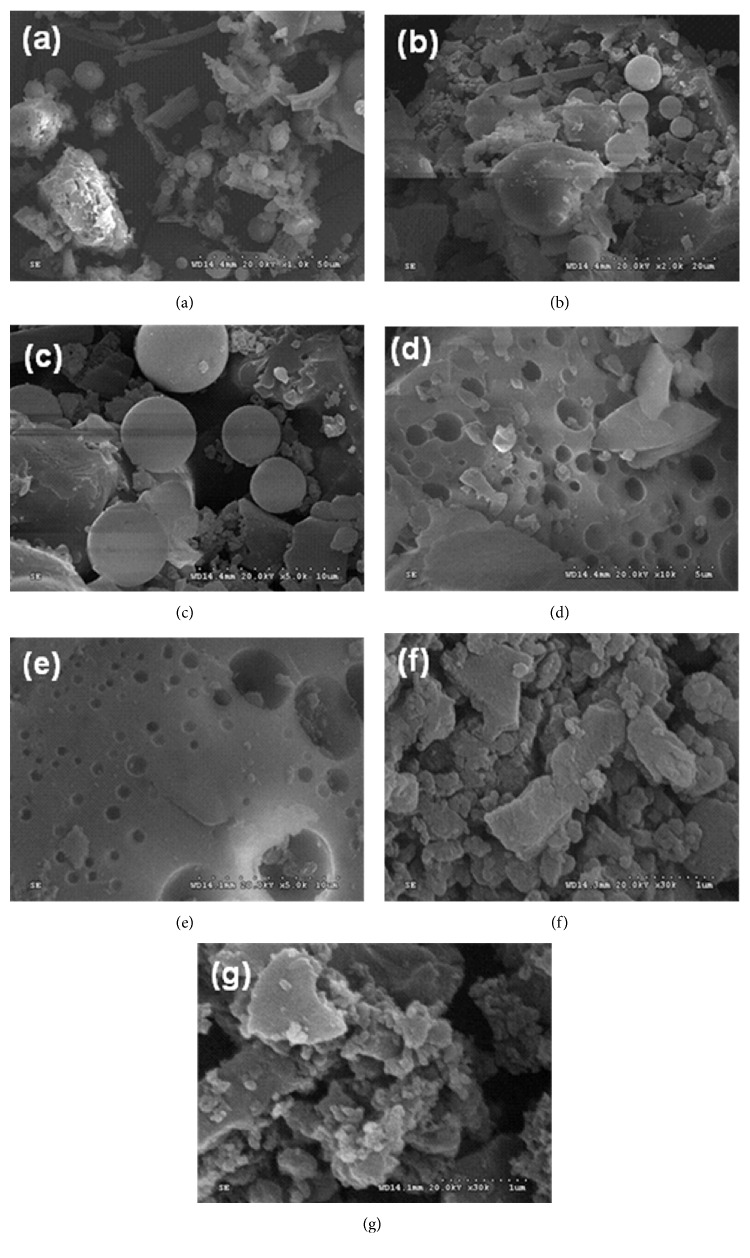
FE-SEM images of polycarbazole ((a)–(d)) E-PCz-*p*-TSA in different magnification, (e) E-PCz, (f) S-PCz-*p*-TSA,and (g) S-PCz.

Morphological studies of PCz samples were carried out by scanning electron microscopy. SEM pictures of E-PCz-*p*-TSA taken at various magnifications are shown in Figure 5. SEM image taken at lower magnifications (Figures 5(a) and 5(b)) shows various shapes such as rod, sphere, and pores. In order to find out the length, width, and diameter of various shapes, SEM image has been taken at higher magnification (Figures 5(c) and 5(d)). Rod was obtained in 18–30 *µ*m of length and 2.5 *µ*m of width, sphere with 3 to 7 *µ*m diameter, and size of the pores 0.6 to 1.1 *µ*m. On conversion of E-PCz-*p*-TSAto its base (E-PCz), SEM images (Figure 5(e)) show only porous structure with small pores of size (0.5 to 1 *µ*m) and higher pores of size (5 to 6.5 *µ*m), whereas SEM images of S-PCz-*p*-TSA (Figure 5(f)) and its corresponding base (Figure 5(g)) show flake-like structure.

### 3.7. XRD Patterns of Polycarbazole

**Figure 6:**
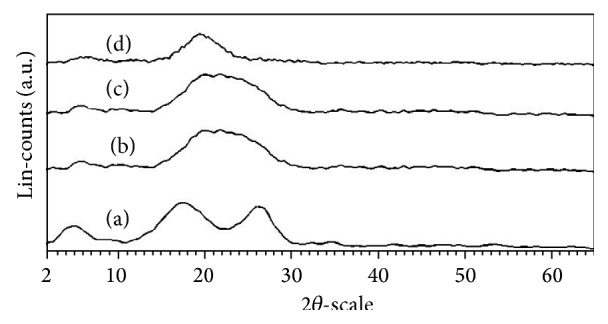
X-ray diffraction patterns of (a) S-PCz-*p*-TSA, (b) S-PCz (c) E-PCz-*p*-TSA, and (d) E-PCz.

X-ray diffraction profiles registered for polycarbazole samples, S-PCz-*p*-TSA, S-PCz, E-PCz-*p*-TSA, and E-PCz, are shown in Figure 2. X-ray diffraction patter of S-PCz-*p*-TSA showed three broad peaks around 2*θ* = 6, 18, and 27° with corresponding* d*-spacing 16.7, 5.1, and 3.3, respectively (Figure 6(a)), whereas PCz samples of S-PCz, E-PCz-*p*-TSA, and E-PCz showed two very broad bands around 6 to 8 and 18 to 23° (Figures 6(b)–6(d)). X-ray results of PCz salts and bases prepared in the present work show amorphous nature.

### 3.8. Thermograms of Polycarbazole

**Figure 7:**
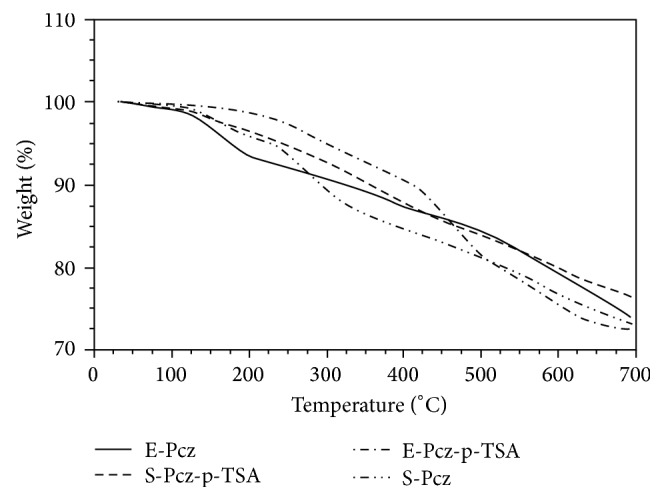
TGA thermograms of Polycarbazole.

TGA thermograms of polycarbazole samples are recorded from ambient temperature to 700°C under nitrogen atmosphere and are shown in Figure 7. TGA thermogram of S-PCz-*p*-TSA, its base (S-PCz), and E-PCzshows that these samples are stable up to 110°C. However, polycarbazole sample prepared by emulsion polymerization pathway (E-PCz-*p*-TSA) is stable up to 200°C.

### 3.9. Cyclic Voltammogram of S-PCz-*p*-TSA

**Figure 8:**
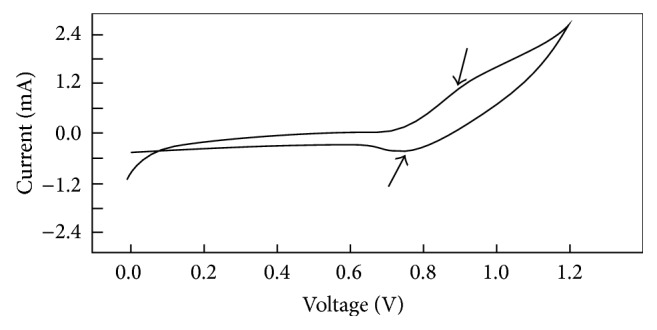
CV of S-PCz-*p*-TSA in acetonitrile using 0.1 M tetraethylammonium tetrafluoroborate as supporting electrolyte at a scan rate 10 mV/s using Ag/AgCl reference electrode.

S-PCz-*p*-TSA powder sample was pressed on stainless steel mesh and used as working electrode for CV measurement. CV of S-PCz-*p*-TSA polymer electrode was carried out in acetonitrile using 0.1 M tetraethylammonium tetrafluoroborate as supporting electrolyte at a scan rate 10 mV/s with Ag/AgCl reference electrode in single one cycle. CV of S-PCz-*p*-TSA shows one redox peak, anodic peak at 0.9 V, and cathodic peak at 0.74 V (Figure 8), which justifies the formation of the polymer. A similar result was observed for polycarbazole prepared by interfacial pathway using ammonium persulfate oxidant by Gupta and Prakash [21]. However, the electroactive nature of S-PCz-*p*-TSA sample is less and this may be due to overoxidation (overoxidation is supported by infrared spectrum).

## 4. Conclusions

Benzoyl peroxide has been demonstrated as oxidizing agent in the oxidation of carbazole to solvent soluble polycarbazole. Polycarbazole salt prepared by solution polymerization pathway was obtained in semiconductor range (1.5 × 10^−5^ S/cm) with density (1.15 g/cm^3^) and reasonable yield (69 wt% with respect to the amount of carbazole used). Polycarbazole prepared by solution and emulsion polymerization pathways gave amorphous powder. Polycarbazole salts prepared by both solution (~10 repeating units) and emulsion polymerization pathways (~12 repeating units) were soluble in less and high polar solvents. Moreover, polycarbazole salt prepared by emulsion polymerization pathway was also soluble in chloroform. Polycarbazole salt prepared by emulsion polymerization pathway shows mixture of shapes with microrod (8–30 *µ*m of length and 2.5 *µ*m of width), sphere (3 to 7 *µ*m diameter), and pores (0.6 to 1.1 *µ*m). Its corresponding base shows only micropores of size 0.5 to 6.5 *µ*m, whereas polycarbazole salt and its corresponding base prepared by solution polymerization pathway show flake-like morphology. Higher thermal stability was obtained for polycarbazole salt prepared by emulsion polymerization pathway than that of the salt prepared by solution polymerization pathway. This polycarbazole powder will be useful for coating and making antistatic films with common insulating polymers.

## Figures and Tables

**Table 1:** Yield, conductivity, and density of the polycarbazole salt (S-PCz-*p*-TSA).

Entry	Reaction conditions	Variation	Yield^*^(%)	Conductivity × 10^−6^ (S/cm)	Density (g/cm^3^)
1	Carbazole = 1.67 g	PTSA (g)			
	BPO = 3 g	1.9	55	5.0	1.15
	Time = 4 h	3.8	52	6.8	1.11

2	Carbazole = 1.67 g	Time (h)			
	PTSA = 3.8 g	4	52	6.8	1.11
	BPO = 3 g	8	54	6.9	1.15
		24	67	0.6	1.17

3	Carbazole = 1.67 g	BPO (g)			
	PTSA = 3.8 g	1.2	34	3.3	1.18
	Time = 4 h	2.4	47	4.6	1.16
		3.0	52	6.8	1.11
		3.6	69	15.1	1.18
		4.8	65	1.4	1.13

^*^Percentage yield of polycarbazole with respect to the weight of carbazole used.

**Table 2:** Infrared spectral peaks (cm^−1^) of polycarbazoles.

	E-PCz-*p*-TSA	E-PCz	S-PCz-*p*-TSA	S-PCz
N–H str.	3405	3410	3405	3405
Aromatic C–H str.	3050	3050	3045	3050
Impurity/supertone bands shifted aromatic C–H str./aliphatic C–H str.	2920	2925	2925	2925
*δ*-OH of moisture (or) C=O str. due to overoxidation	1730	Small peak	1725	NIL
Aromatic ring str.	1600	1600	1600	1605
	1450	1450	1455	1455
C–N str.	1390	1390	1395	1385
	1320	1320	1320	1320
C–N vibration	1235	1230	1235	1230
Due to S=O group	1165	Small peak	1120	V.small
	1060	Small peak	1060	V.small
C–H def. of trisubstituted benzene ring	885	885	880	875
	805	805	805	800
	745	745	745	740
